# Influence of Micronutrients on the Food Consumption Rate and Silk Production of *Bombyx mori* (Lepidoptera: Bombycidae) Reared on Mulberry Plants Grown in a Mountainous Agro-Ecological Condition

**DOI:** 10.3389/fphys.2019.00878

**Published:** 2019-07-09

**Authors:** Lalfelpuii Ruth, Souvik Ghatak, Sarathbabu Subbarayan, Bidyut Nath Choudhury, Guruswami Gurusubramanian, Nachimuthu Senthil Kumar, Tang Bin

**Affiliations:** ^1^Department of Biotechnology, Mizoram University, Aizawl, India; ^2^Regional Office, Central Silk Board, Guwahati, India; ^3^Department of Zoology, Mizoram University, Aizawl, India; ^4^College of Life and Environmental Sciences, Hangzhou Normal University, Hangzhou, China

**Keywords:** bivoltine, *Bombyx mori*, food consumption, micronutrients, economics, mulberry

## Abstract

The study involves analyzing the performance of bivoltine *Bombyx mori* larvae reared on different host plants varieties. The consumption rate (CR) of different strains of *B. mori* was high when fed with Jorhat and TR10 mulberry plant varieties. Jorhat and TR10 mulberry plant varieties were found to contain significant amount of calcium, potassium, magnesium and phosphorus. Local (Hmute) mulberry plant variety had high amount of protein, carbohydrate and reducing sugar. Majority of the *B. mori* strains reared on Jorhat and TR10 mulberry plant varieties had high level of fibroin protein which resulted in increased silk productivity than those larvae reared with other mulberry varieties. The filament length was higher when reared on Jorhat and TR10 mulberry plant varieties. CSR4 × CSR2, FC1 × FC2, and FC2 × FC1 strains reared on Jorhat and TR10 mulberry plant varieties performed well in terms of economic parameters. Proteins and other nutrients in combination with high levels of micronutrients are very much essential for better silk quality. The present study attempted to identify the most suitable host plants for silkworm rearing under mountainous agro-ecological conditions which can lead to sustainable production of silk in relation to physiological and economic parameters.

## Introduction

Sericulture is a sustainable, eco-friendly and agro-forestry oriented trade comprising cultivation of mulberry plant varieties, rearing of silkworms, and production of silk. It is one of the most labor-intensive sectors and has played a critical role in rural development and economic growth. Most of the marketable silk around the world is being produced from the mulberry silkworm, *Bombyx mori* L. (Yogananda-Murthy et al., 2013). *B. mori* is an essentially monophagous and host plant-specific insect that feeds solely on mulberry leaves (*Morus alba*, Family: Moracea) (Savithri et al., 2013). Two kinds of silk proteins have been distinguished as major components of silk cocoons, the first being fibroin – a fibrous protein secreted in the lumen of the posterior silk gland of *B. mori* and the second being sericin, a natural macromolecular protein that serves as an adhesive to unite fibroin for making silk cocoons of silkworm, *B. mori* (Sabina et al., 2012).

India has rich resources of mulberry varieties that are traditionally cultivated, and a few exotic varieties have been introduced from time to time. Besides the influence of environmental factors, the silk productivity is related to the quantity and quality of mulberry leaves (Nagaraju, 2002). Nutritional physiology has a vital role in influencing the performances of different stages of silkworm. To better understand the chemical ecology of the insect-plant relationship, studying the quantitative aspect of nutrition in the insect is important (Waldbauer, 1968). Development of silkworm is greatly influenced by the nutrient composition of the mulberry host leaves, which is also the determining factor of the quality of silk (Jyothi et al., 2014). The performance of silkworm is evident by the digestion and assimilation of the nutritional materials present in mulberry leaves (Lalfelpuii et al., 2014a,b). The life cycle routine of the silkworm from identical genetic stock varies significantly based on nutritional quality of mulberry leaves (Rahmathulla, 2011). As such, the amount of food consumed and the quantity digested by the silkworms have a direct effect on its physiological performance and silk production. Pioneering research has proved that deficiency of certain nutrients or imbalance of nutrient on the diet affects the digestibility and metabolic activity of larvae (Waldbauer, 1964).

Several scientists have established variations in biochemical components of mulberry leaves. Bose et al. (1991) and Neog et al. (2011) revealed that mulberry varieties differ significantly in the composition of nutrients. The protein content (soluble and crude) of mulberry leaves is the paramount nutritional factor in determining the life cycle performance of silkworm (Pillai and Jolly, 1985). Machii and Katagiri (1991) indicated that the nutritive value of mulberry leaves depends on nitrogen and amino acid contents. Carbohydrates are very important for maintaining healthy growth of young silkworm larvae. Fats or lipids are particularly the main forms of energy reserves and are important for the proper development of wild silkmoth, *Antheraea assama* (Kataky and Hazarika, 1997). These studies have mainly focused on the growth and reproduction of the silkworm. Different mulberry varieties react to climatic conditions, particularly temperature, which affects their quality (Lalfelpuii et al., 2014b). To select a silkworm strain for its commercial exploitation, attributes like geographical environment, viability to rear, silk traits, etc., are to be taken into consideration. Therefore, it is required that strains are selected for particular geographical environments by utilizing the races acclimatized to that location (Lalfelpuii et al., 2014a). However, not much research has been done to understand the role of nutrients on the economic parameters, especially in a high-altitude hilly mountainous agro-climatic zone.

The amount of food consumed and the quantity digested by the silkworms will have direct effects on its performance, mating success and reproduction. Deficiency of certain nutrients or a nutritionally imbalanced diet affects the digestibility and metabolic activity of larvae (Waldbauer, 1964). Therefore, we would expect to find that different silkworm strains performed best on its most preferred host plants (Slansky and Scriber, 1985). The rearing performance of silkworm races are described in terms of larval weight and improved economic traits like cocoon weight, shell weight, and silk percentage (Gangawar, 2010; Seidavi, 2011). Cocoon weight and shell weight are the most important characteristics evaluated for productivity (Gaviria et al., 2006).

In the present study, an attempt has been made to assess the performance of *B. mori* on four mulberry varieties (Jorhat, TR10, BC2-59, and the local Hmute) to identify the most suitable host plant with regard to the economic traits of different bivoltine silkworm strains in the Eastern Himalayan region of Northeast India.

## Materials and Methods

### Mulberry Cultivation and Study Site

Cuttings of mulberry varieties viz. Jorhat, TR10, and BC2-59 were procured from Research Extension Centre, Central Silk Board, Shillong, India. The local (Hmute) mulberry variety was collected from Aizawl, Northeast India. Humte is found extensively in the hills and jungles of Mizoram in wild conditions, and it possesses comparatively high adaptability to Mizoram agro-climatic conditions (Supplementary Table 1). The selected mulberry varieties were grown in the experimental field of the Department of Biotechnology, Mizoram University, Aizawl (altitude of 950 MSL; Longitude 92°38^′^ to 92°42^′^E; Latitude 23°42^′^ to 23°46^′^N). Aizawl has mild climate temperatures ranging from 20 to 30°C in summer and 3 to 20°C in winter. It rains heavily from May to September, with little rain in the dry (cold) season, and the climate pattern is moist tropical to moist sub-tropical with average state rainfall of 215 cm and precipitation of 85.09 cm per annum. Cultivation of mulberry plant varieties was done as per the agronomic practices followed for rain-fed mulberry accessions (Kant and Bhat, 2010).

### Mass Culture of Silkworm

Disease-free layings of six bivoltine silkworm strains – SK6 × SK7, SK7 × SK6, CSR2 × CSR4, CSR4 × CSR2, FC1 × FC2, and FC2 × FC1 – were procured from germplasm of National Silkworm Seed Organization, Bangalore, India and one pure Japanese strain J112 (S7) was procured from germplasm of the Department of Sericulture, Aizawl, India.

Silkworm rearing was done as per standard rearing package (Krishnaswami, 1978). All seven bivoltine races of *B. mori* were reared with locally available mulberry leaves (Hmute) up to second instar to initiate and uniformly stabilize the cultures before starting the experiments on different host plant leaves. From the third instar active feeding stage onward, the larvae were individually fed with four different mulberry varieties (temperature 25°C and relative humidity 72%) until the end of the larval instars. There were 84 experimental sets, and each experimental set comprised of a rearing tray with 20 larvae (7 silkworm races × 4 host plant varieties × 3 replicates = 84 sets × 20 larvae = 1680 larvae). The larvae were fed *ad libitum* four times daily (4 h interval) with chopped mature leaves obtained from the newly formed twigs on the top of the mulberry plants (Supplementary Figure 1). The initial and final weights (leftover) of the leaves were measured. The leaves were collected every day at 06:00 h and stored in moist gunny bags. The first feed was given at 07:00 h while the last feeding was at 19:00 h, and this routine was continued until cocoon formation. Nets with meshes were laid on the top of the rearing tray, and fresh leaves were spread above them. The tray was cleaned daily and the excreta and leftover food was discarded. The larvae were dusted with bleaching powder (3%) before the first feed of every instar (Krishnaswami, 1978).

### Macro- and Micronutrient Analysis of Mulberry Plants

A total of 100 mg of fresh mature mulberry leaves of different host plants were subjected to biochemical analysis. Quantitative estimation of total proteins (Lowry et al., 1951), total carbohydrates (Yemm and Willis, 1954), total lipids (Bligh and Dyer’s, 1959), total amino acids (Moore and Stein, 1948) and total reducing sugar by the Dinitrosalicylic method (Miller, 1972) were measured. Moisture content of mulberry leaves was measured by computing the difference between initial and final dried weight (incubated at 37°C) and represented in percentage (Shobana et al., 2010).

For micronutrient analysis, mature leaves of the four mulberry varieties were washed with distilled water, shade dried at room temperature and ground to powder using a mixer grinder. Wet digestion was performed to estimate the level of nutrients. An accurately weighed leaf powder (0.3 g) was transferred to closed flask. A total of 5 mL of 30% Hydrogen peroxide and 10 mL of concentrated sulphuric acid solutions was added and the sample was digested until the solution became clear. After digestion, the sample was diluted to 100 mL with distilled water in a volumetric flask. The samples were used for analysis of minerals such as magnesium, calcium, potassium, and phosphorus (Jackson, 1973).

### Food Utilization Indices

A food utilization experiment was performed using 84 experimental sets, and each experimental set comprised of a rearing tray with 20 larvae (7 silkworm races × 4 host plant varieties × 3 replicates = 84 sets × 20 larvae = 1680 larvae). Larvae were maintained under controlled conditions (temperature: 25 ± 2°C; moisture content 70–80%; light: dark 12:12 h), and weight of the larva, food consumed and feces produced were measured daily. The host plant feed was changed daily and the fresh as well as leftover leaves were weighed. The experiment was continued for 6 days, and observations were recorded every 24 h. Food utilization indices (all based on dry weight) were calculated (Waldbauer, 1968; Slansky and Scriber, 1985):

**Table:** 

Growth rate (GR)	= P/T
Consumption rate (CR)	= E/T
Consumption index (CI)	= E/TA
Approximate digestibility (AD) (%)	= 100 (E - F)/E
Efficiency of conversion of ingested food (ECI) (%)	= 100P/E
Efficiency of conversion of digested food (ECD) (%)	= 100P/(E - F)



Where, A: mean dry weight of animal during T; E: dry weight of food eaten; F: dry weight of feces produced; P: dry weight gain of insects; T: duration of the experimental period.

### Silkworm Larva Performance on Different Diets

The weights of larva, cocoon, shell, and filament were measured using electronic balance (±0.01 g; Mettler, United States). Shell percentage and denier were calculated using the formula: Shell (%) = Cocoon shell weight/cocoon weight × 100; Denier = [(Filament weight (g)/filament length (meter)] × 9000. The length of the silk thread (bave) from the cocoon was reeled using a reeling apparatus (eprouvette) and was measured in meters. The silk filament length represents the percentage of silk by measuring the length of the bave contained in the shell (FAO, 1999).

### Biochemical Estimation of *B. mori*

The silkworm larva that fed on different host plants were used for estimation of biochemical parameters. Larval hemolymph and silk gland proteins were estimated using Lowry et al. (1951). Fibroin and sericin percentage were analyzed from cocoon (Thirumalaisamy et al., 2009). Enzyme assay from midgut tissue extracts were performed by dissecting the midgut. A total of 25 mg of the midgut was homogenized (200 μL phosphate buffered saline) and centrifuged at 3000 rpm for 10 min. Enzyme analysis was performed using this supernatant. Quantitative amylase activity of different midgut samples was analyzed using standard protocol (Nagaraju and Abraham, 1995). Mid gut (50 μL) samples were mixed with citrate buffer (50 μL) and allowed to incubate at 37°C for 3 min and the reaction was inhibited by adding 3, 5-Dinitrosalicylic acid (200 μL). The reaction mixture was kept in boiling temperature in a water bath, and the color developed was read at 540 nm. Protease activity of different midgut samples was analyzed using standard protocol (Eguchi and Iwamoto, 1976). 30 μL of 1% casein was taken in centrifuge tubes followed by 30 μL of 0.1 M borate buffer (pH 11.0) and 60 μL of 10% Trichloroacetic acid. To this, 50 μL of the samples was added and centrifuged at 3000 rpm for 10 min. The sample mixture (200 μL) was mixed with sodium hydroxide (20 μL, 0.5 N) and folin-ciocalteu reagent (50 μL) and incubated for 30 min. The absorbance was measured at 660 nm.

### Statistical Analysis

All data on food consumption and biochemical analysis were affirmed as mean ± SE by using statistical software OriginPro 8 v8.0724, Northampton, MA, United States. The normality distribution of the variables was tested using one sample Kolmogorov–Smirnov test. Differences in measured parameters among the groups were analyzed by a one-way analysis of variance (ANOVA) test due to normal distribution. The results were analyzed by ANOVA followed by a Duncan test for *post hoc* comparisons, and class predictions were computed by R statistical package (at *P* < 0.05) (Duncan, 1955).

## Results

### Biochemical Parameters of Mulberry Host Plants

There were differences in the levels of biochemical components among the tested mulberry cultivars (Table 1). Total protein content varied significantly between the different mulberry varieties (Hmute > TR10 > BC2-59 > Jorhat). Lipid content was highest in the Jorhat mulberry variety and lowest in BC2-59. Carbohydrate content was high in the Hmute variety and low in the BC2-59 variety. Total amino acid, water content, potassium, and calcium were highest in TR10 and lowest in the BC2-59 mulberry plant variety. Magnesium and phosphorus were high in the Jorhat mulberry plant variety.

**Table 1:** Biochemical parameters of mulberry plant varieties.

Host plant	Protein (μg/mL)	Amino acid (μg/mL)	Lipid (μg/mL)	Carbohydrate (μg/mL)	Reducing sugar (μg/mL)	Water content	Magnesium (mM/L)	Calcium (mM/L)	Potassium (mM/L)	Phosphorous (mM/L)
Hmute	1766.2 ± 4.72^a^	34.99 ± 1.28^b^	930 ± 2.96^b^	3842.83 ± 4.97^a^	193.23 ± 5.49^a^	1.268 ± 0.73^a^	0.562 ± 0.082^a^	1.362 ± 0.074^b^	1.752 ± 0.086^b^	0.352 ± 0.049^b^
Jorhat	449.4 ± 3.91^d^	34.07 ± 1.03^b^	970 ± 3.61^a^	3839.06 ± 7.36^b^	131.05 ± 9.31^c^	1.244 ± 0.39^a^	0.678 ± 0.079^a^	1.582 ± 0.115^a^	1.793 ± 0.095^b^	1.672 ± 0.064^a^
BC2-59	473.7 ± 3.82^c^	31.47 ± 1.95^b^	880 ± 3.72^c^	3746.09 ± 7.48^c^	164.91 ± 4.47^b^	1.215 ± 0.41^a^	0.529 ± 0.161^a^	1.322 ± 0.108^b^	1.872 ± 0.061^ab^	0.363 ± 0.029^b^
TR10	909.8 ± 5.49^b^	40.58 ± 1.68^a^	930 ± 3.29^b^	3841.56 ± 5.72^a^	168.53 ± 6.28^b^	1.272 ± 0.86^a^	0.645 ± 0.038^a^	1.583 ± 0.093^a^	1.952 ± 0.072^a^	0.371 ± 0.037^b^
*F*-value _3,11_	18391	6.314	116.96	53.995	14.834	0.001732	0.4809	2.004	1.237	197.12
*p*-value	<0.0001	<0.0167	<0.0001	<0.0001	<0.0012	<0.9999	<0.7046	<0.1921	<0.3583	<0.0001


*The data are mean ± standard error (*n* = 3 observations). *p-*values were determined by one-way ANOVA with subsequent Tukey’s *post hoc* test. Values of treatment groups with different alphabetical letters show statistically significant difference at *p* < 0.05 and values with similar letters show no significance at *p* > 0.05. The *F*-value is the ratio of the mean regression sum of squares divided by the mean error sum of squares (variance within samples/variance between samples).*

### Food Utilization Indices

The food consumption and ingestion indices of fifth instar *B. mori* larvae reared on Hmute, Jorhat, BC2-59, and TR10 mulberry plant varieties are shown in Table 2. Consumption Rate (CR), Consumption Index (CI), and Approximate Digestibility (AD) were high in TR10-reared larvae for all the *B. mori* strains tested. The growth rate (GR) was highest in larvae fed Hmute for both the SK6 × SK7 and CSR2 × CSR4 strains. In the FC2 × FC1 strain, the GR and CI were highest in larvae fed with the Jorhat plant variety. Significant variations were observed in the ECI and ECD parameters between the silkworm strains and mulberry varieties. High ECI (22.49%) and ECD (24.55%) were recorded in the SK6 × SK7 strain reared on Hmute mulberry varieties.

**Table 2:** Food utilization efficiency measures of fifth instar larvae of *B. mori* strains reared on different mulberry varieties.

Strain	Host plant	GR (g/larvae/day)	CR (g/larvae/day)	CI (g/larvae/day)	AD (%)	ECI (%)	ECD (%)
**SK6 × SK7**	Hmute	0.076 ± 0.016^a^	0.345 ± 0.086^a^	0.196 ± 0.025^a^	91.58 ± 1.90^a^	22.49 ± 0.42^a^	24.55 ± 0.68^a^
	Jorhat	0.067 ± 0.017^b^	0.331 ± 0.125^a^	0.172 ± 0.045^a^	92.03 ± 2.01^a^	20.24 ± 0.66^b^	21.99 ± 0.78^b^
	BC2-59	0.049 ± 0.015^b^	0.390 ± 0.149^a^	0.164 ± 0.024^a^	91.88 ± 1.59^a^	12.55 ± 0.54^d^	13.66 ± 0.91^c^
	TR10	0.060 ± 0.017^b^	0.521 ± 0.135^a^	0.251 ± 0.040^a^	93.76 ± 1.85^a^	15.43 ± 0.77^c^	12.26 ± 0.64^c^
*p*-value		<0.6982	<0.7099	<0.3460	<0.8356	<0.0001	<0.0001
*F*-Value _3,11_		0.4910	0.4725	1.278	0.2842	54.411	63.853
**SK7 × SK6**	Hmute	0.058 ± 0.015^a^	0.348 ± 0.129^a^	0.162 ± 0.028^a^	90.79 ± 1.84^a^	16.88 ± 0.54^a^	18.59 ± 0.70^a^
	Jorhat	0.067 ± 0.019^b^	0.466 ± 0.137^a^	0.246 ± 0.030^a^	93.20 ± 1.89^a^	14.49 ± 0.78^b^	15.55 ± 0.87^b^
	BC2-59	0.061 ± 0.019^b^	0.388 ± 0.125^a^	0.194 ± 0.060^a^	92.07 ± 1.69^a^	15.77 ± 0.49^ab^	17.13 ± 0.85^ab^
	TR10	0.064 ± 0.016^b^	0.520 ± 0.133^a^	0.263 ± 0.026^a^	94.07 ± 1.34^a^	16.18 ± 0.89^ab^	13.15 ± 0.72^c^
*p*-value		<0.9842	<0.7925	<0.2976	<0.5801	<0.1804	<0.0067
*F*-Value _3,11_		0.04988	0.3470	1.456	0.6960	2.087	8.718
**CSR2 × CSR4**	Hmute	0.096 ± 0.018^a^	0.335 ± 0.147^a^	0.219 ± 0.036^ab^	92.83 ± 1.76^a^	28.65 ± 0.63^a^	30.87 ± 0.82^a^
	Jorhat	0.046 ± 0.006^ab^	0.458 ± 0.168^a^	0.164 ± 0.062^b^	94.65 ± 1.76^a^	10.17 ± 0.59^d^	10.74 ± 0.65^c^
	BC2-59	0.059 ± 0.008^ab^	0.391 ± 0.128^a^	0.184 ± 0.025^b^	91.83 ± 1.64^a^	15.28 ± 0.65^c^	16.64 ± 0.68^b^
	TR10	0.083 ± 0.019^b^	0.525 ± 0.136^a^	0.325 ± 0.036^a^	93.57 ± 1.22^a^	19.63 ± 0.69^b^	16.88 ± 0.96^b^
*p*-value		<0.1234	<0.8110	<0.1008	<0.6643	<0.0001	<0.0001
*F*-Value _3,11_		2.612	0.3199	2.912	0.5465	149.30	117.79
**CSR4 × CSR2**	Hmute	0.068 ± 0.016^b^	0.348 ± 0.125^a^	0.192 ± 0.024^a^	90.58 ± 1.67^a^	19.72 ± 0.87^ab^	21.77 ± 0.67^a^
	Jorhat	0.241 ± 0.018^a^	0.452 ± 0.126^a^	0.171 ± 0.029^a^	94.82 ± 1.97^a^	5.33 ± 0.88^c^	5.62 ± 0.95^c^
	BC2-59	0.078 ± 0.015^b^	0.384 ± 0.127^a^	0.224 ± 0.046^a^	92.17 ± 1.77^a^	20.48 ± 0.49^a^	22.22 ± 0.73^a^
	TR10	0.068 ± 0.015^b^	0.514 ± 0.129^a^	0.285 ± 0.052^a^	94.47 ± 1.67^a^	17.93 ± 0.58^b^	14.15 ± 0.63^b^
*p*-value		<0.0001	<0.7991	<0.2677	<0.3476	<0.0001	<0.0001
*F*-Value _3,11_		28.035	0.3373	1.584	1.273	95.777	107.04

**Strain**	**Host plant**	**GR (g/insect/day)**	**CR (g/insect/day)**	**CI (g/insect/day)**	**AD (%)**	**ECI (%)**	**ECD (%)**

**FC1 × FC2**	Hmute	0.063 ± 0.017^a^	0.355 ± 0.149^a^	0.176 ± 0.043^b^	89.90 ± 2.01^a^	17.91 ± 0.84^b^	19.93 ± 0.91^b^
	Jorhat	0.049 ± 0.007^a^	0.471 ± 0.137^a^	0.279 ± 0.026^ab^	92.68 ± 1.56^a^	10.45 ± 0.76^c^	11.28 ± 0.73^c^
	BC2-59	0.078 ± 0.016^a^	0.395 ± 0.126^a^	0.268 ± 0.038^ab^	91.86 ± 1.95^a^	19.75 ± 0.57^ab^	21.50 ± 0.96^b^
	TR10	0.081 ± 0.016^a^	0.523 ± 0.157^a^	0.334 ± 0.039^a^	93.92 ± 1.94^a^	20.29 ± 0.85^a^	39.37 ± 0.89^a^
*P-*value		<0.4306	<0.8394	<0.0875	<0.5223	<0.0001	<0.0001
*F*-Value _3,11_		1.027	0.2787	3.129	0.8116	35.514	180.83
**FC2 × FC1**	Hmute	0.071 ± 0.016^a^	0.357 ± 0.126^a^	0.195 ± 0.061^a^	89.92 ± 1.98^b^	20.00 ± 0.85^a^	22.25 ± 0.69^a^
	Jorhat	0.078 ± 0.013^a^	0.471 ± 0.129^a^	0.282 ± 0.050^a^	92.90 ± 1.94^ab^	16.72 ± 0.67^b^	18.00 ± 0.89^b^
	BC2-59	0.076 ± 0.017^a^	0.395 ± 0.129^a^	0.240 ± 0.025^a^	91.27 ± 1.89^b^	19.34 ± 0.78^a^	21.19 ± 0.80^a^
	TR10	0.062 ± 0.015^a^	0.525 ± 0.128^a^	0.279 ± 0.027^a^	93.69 ± 1.44^a^	16.83 ± 0.69^b^	12.58 ± 0.90^c^
*p-*value		<0.8820	<0.7924	<0.4921	<0.5038	<0.0293	<0.0001
*F*-Value _3,11_		0.2169	0.3471	0.8776	0.8516	5.086	27.761
**J112**	Hmute	0.067 ± 0.018^c^	0.406 ± 0.128^a^	0.215 ± 0.028^a^	61.47 ± 1.23^c^	16.58 ± 0.86^c^	26.98 ± 0.98^d^
	Jorhat	0.131 ± 0.015^a^	0.492 ± 0.126^a^	0.447 ± 0.139^a^	66.38 ± 1.88^b^	26.62 ± 0.85^a^	40.10 ± 0.67^a^
	BC2-59	0.077 ± 0.019^bc^	0.400 ± 0.087^a^	0.236 ± 0.034^a^	59.08 ± 1.45^c^	19.42 ± 0.69^b^	32.88 ± 0.79^b^
	TR10	0.118 ± 0.019^ab^	0.552 ± 0.066^a^	0.461 ± 0.135^a^	70.69 ± 1.01^a^	25.23 ± 0.75^a^	30.39 ± 0.95^c^
*p-*value		<0.0931	<0.0931	<0.2298	<0.0019	<0.0019	<0.0001
*F*-Value _3,11_		3.033	0.7038	1.774	13.117	36.152	42.143


*GR (Growth rate); CR (Consumption rate); CI (Consumption index); AD (Approximate digestibility); ECI (Efficiency of conversion of ingested food); ECD (Efficiency of conversion of digested food). The data are mean ± standard error (*n* = 3 observations). *p-*values were determined by one-way ANOVA with subsequent Tukey’s *post hoc* test. Values of treatment groups with different alphabetical letters show statistically significant difference at *p* < 0.05, and values with similar letters show no significance at *p* > 0.05. The *F*-value is the ratio of the mean regression sum of squares divided by the mean error sum of squares (variance within samples/variance between samples).*

### Economic Performance of *B. mori*

**FIGURE 1:**
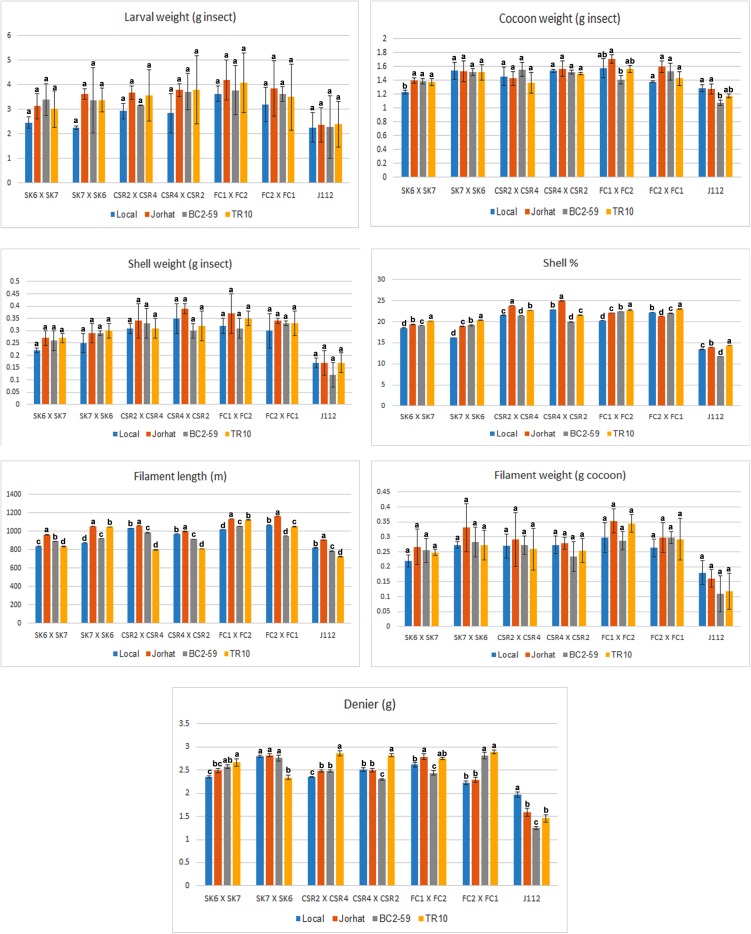
Economic parameters of *B. mori* strains reared on different mulberry varieties. Six bivoltine silkworm strains were used – SK6 × SK7, SK7 × SK6, CSR2 × CSR4, CSR4 × CSR2, FC1 ×FC2, FC2 × FC1 – as well as one multivoltine strain, J112. Host plants used – Hmute, Jorhat, BC2-59, and TR10. Data are presented as mean ± standard error mean (*n* = 1680 larvae/treatment). There were 84 experimental sets and each experimental set comprised of a rearing tray with 20 larvae (7 silkworm races × 4 host plant varieties × 3 replicates = 84 sets × 20 larvae = 1680). Statistical comparison was performed using one-way ANOVA followed by Tukey’s *post hoc* tests for all pair-wise multiple comparisons. Bars with different letters (a, b, c, d) indicate that treatment groups are significantly different at *P* < 0.05 and with same letters indicate that treatment groups are not statistically significant at *P* > 0.05.

The silkworm economic parameters such as cocoon weight, shell weight, and filament length were high on larvae fed with the Jorhat mulberry plant variety in all the *B. mori* strains tested. A higher value of Shell percentage was evident on all the *B. mori* strains reared with the TR10 mulberry plant variety except CSR2 × CSR4 and CSR4 × CSR2. Filament length was higher when the larvae were reared with the Jorhat mulberry plant variety (Figure 1).

The highest shell weight and shell percentage were found in the SK7 × SK6 strain reared on TR10. Filament length and weight were higher when the larvae were reared with the Jorhat plant variety. In the CSR2 × CSR4 strain, the shell weight as well as filament length were high on the Jorhat plant variety-reared larvae. Denier was higher on the TR10 mulberry plant variety. The shell weight and filament length were higher in the CSR4 × CSR2 strain reared with the Jorhat mulberry plant variety. The larval weight of the FC1 × FC2 strain was highest on Jorhat followed by the TR10 mulberry plant variety. The FC2 × FC1 strain had high larval, cocoon, shell, and filament weight in larvae reared on the Jorhat mulberry plant variety. The larval strains reared on the TR10 mulberry plant variety exhibited high Denier in all the larval strains except SK7 × SK6 and J112 (Figure 1).

### Biochemical and Enzymatic Activities of *B. mori* Strains

Silk is mainly composed of two proteins, fibroin and sericin, accounting for about 70 and 30% of total silk cocoon weight, respectively. The SK6 × SK7 cocoon had high fibroin (74.0%) and sericin (26.0%) contents when reared on the Jorhat mulberry variety. The CSR2 × CSR4 strain cocoon had 71.01 and 28.99% of fibroin and sericin, respectively, when reared on Jorhat mulberry plant variety. The silkworm strains FC1 × FC2 and SK7 × SK6 had a high fibroin to sericin ratio when reared on the TR10 host plant (Table 3). TR10 mulberry plant variety-reared larvae had highest hemolymph protein among all the host plants tested in the present study. The highest silk gland protein content was observed in larvae reared on the BC2-59 mulberry plant variety. Maximum amylase activity was found in those larval midgut samples reared on the Jorhat mulberry variety (Table 3).

**Table 3:** Biochemical and Enzymatic profiles of *B. mori* strains reared on different host plants.

Strain	Host plant	Fibroin % (Cocoon)	Sericin % (Cocoon)	Hemolymph protein (μg/mL)	Silk gland protein (μg/mL)	Amylase (μg/mL)	Protease (μg/mL)
**SK6 × SK7**	Hmute	70.30 ± 0.05^c^	29.70 ± 0.04^b^	416.44 ± 0.88^b^	365 ± 1.20^b^	1.01 ± 0.08^b^	0.20 ± 0.08^a^
	Jorhat	74.00 ± 0.12^a^	26.00 ± 0.07^d^	472.61 ± 0.90^a^	525 ± 2.23^a^	1.38 ± 0.06^a^	0.20 ± 0.04^a^
	BC2-59	72.32 ± 0.09^b^	27.68 ± 0.06^c^	416.20 ± 1.30^b^	332 ± 1.85^c^	0.60 ± 0.04^c^	0.06 ± 0.07^a^
	TR10	66.37 ± 0.05^d^	33.63 ± 0.07^a^	414.91 ± 0.96^b^	282 ± 1.78^d^	0.61 ± 0.03^c^	0.20 ± 0.05^a^
*P*-value		<0.0001	<0.0001	<0.0001	<0.0001	<0.0001	<0.3475
*F*-Value _3,11_		1571.6	2881.3	768.23	3393.3	44.437	1.273
**SK7 × SK6**	Hmute	74.47 ± 0.08^c^	25.53 ± 0.06^c^	407.37 ± 1.20^b^	354 ± 1.30^b^	0.93 ± 0.03^a^	0.19 ± 0.01^b^
	Jorhat	65.62 ± 0.07^d^	34.38 ± 0.04^a^	363.17 ± 0.81^c^	425 ± 1.85^a^	1.02 ± 0.06^a^	0.28 ± 0.02^a^
	BC2-59	74.29 ± 0.06^b^	25.71 ± 0.03^b^	336.83 ± 1.57^d^	348 ± 1.42^c^	0.62 ± 0.08^b^	0.05 ± 0.03^c^
	TR10	76.04 ± 0.03^a^	23.96 ± 0.04^d^	410.61 ± 0.75^a^	256 ± 1.56^d^	1.05 ± 0.02^a^	0.26 ± 0.02^a^
*P*-value		<0.0001	<0.0001	<0.0001	<0.0001	<0.0016	<0.0016
*F*-Value _3,11_		5646.2	11586	997.23	2009.1	13.699	24.074
**CSR2 × CSR4**	Hmute	62.74 ± 0.10^d^	37.26 ± 0.01^a^	329.57 ± 0.89^d^	380 ± 3.01^b^	0.72 ± 0.06^b^	0.19 ± 0.04^a^
	Jorhat	71.01 ± 0.04^a^	28.99 ± 0.14^d^	376.17 ± 0.78^c^	345 ± 1.03^c^	0.95 ± 0.05^a^	0.19 ± 0.05^a^
	BC2-59	68.60 ± 0.03^b^	31.40 ± 0.07^c^	395.54 ± 0.85^b^	511 ± 1.29^a^	0.66 ± 0.09^b^	0.07 ± 0.07^a^
	TR10	67.08 ± 0.10^c^	32.92 ± 0.08^b^	484.79 ± 0.63^a^	272 ± 2.05^d^	0.62 ± 0.09^b^	0.09 ± 0.06^a^
*P*-value		<0.0001	<0.0001	<0.0001	<0.0001	<0.0548	<0.3390
*F*-Value _3,11_		2150.1	1560.6	6714.0	2503.1	3.903	1.302
**CSR4 × CSR2**	Hmute	68.83 ± 0.09^c^	31.17 ± 0.07^b^	425.98 ± 1.76^c^	425 ± 1.05^b^	0.74 ± 0.03^a^	0.20 ± 0.03^a^
	Jorhat	94.97 ± 0.08^a^	5.03 ± 0.06^d^	287.49 ± 0.84^d^	348 ± 1.68^c^	0.67 ± 0.05^ab^	0.08 ± 0.02^b^
	BC2-59	67.47 ± 0.13^d^	32.53 ± 0.16^a^	438.57 ± 0.64^b^	611 ± 2.54^a^	0.61 ± 0.03^b^	0.09 ± 0.04^b^
	TR10	69.35 ± 0.05^b^	30.65 ± 0.10^c^	488.10 ± 0.59^a^	427 ± 1.89^b^	0.71 ± 0.04^ab^	0.06 ± 0.02^b^
*P*-value		<0.0001	<0.0001	<0.0001	<0.0001	<0.1732	<0.0338
*F*-Value _3,11_		20665	15885	6484.1	3579.7	2.141	4.798
**FFC1 × FC2**	Hmute	68.83 ± 0.09^b^	31.17 ± 0.07^b^	424.98 ± 1.76^b^	425 ± 1.05^b^	0.71 ± 0.01^a^	0.20 ± 0.03^a^
	Jorhat	79.25 ± 0.07^d^	20.75 ± 0.05^c^	482.41 ± 0.90^a^	422 ± 1.09^b^	0.73 ± 0.02^a^	0.08 ± 0.05^b^
	BC2-59	68.16 ± 0.04^c^	31.84 ± 0.05^a^	393.81 ± 0.95^c^	624 ± 1.65^a^	0.70 ± 0.03^a^	0.17 ± 0.04^ab^
	TR10	83.91 ± 0.07^a^	16.09 ± 0.04^d^	425.27 ± 0.79^b^	282 ± 2.06^c^	0.74 ± 0.04^a^	0.07 ± 0.09^b^
*p*-value		<0.0001	<0.0001	<0.0001	<0.0001	<0.7278	<0.3446
*F*-Value _3,11_		12465	21136	1004.4	8549.7	0.4444	1.282
**FC2 × FC1**	Hmute	71.20 ± 0.04^c^	28.80 ± 0.10^b^	385.21 ± 2.64^b^	480 ± 2.20^b^	0.61 ± 0.13^a^	0.08 ± 0.02^b^
	Jorhat	71.92 ± 0.02^b^	28.08 ± 0.03^c^	389.47 ± 0.73^b^	478 ± 1.07^b^	1.27 ± 0.48^a^	0.27 ± 0.05^a^
	BC2-59	74.00 ± 0.05^a^	26.00 ± 0.02^d^	263.04 ± 2.00^c^	619 ± 3.09^a^	0.61 ± 0.18^a^	0.09 ± 0.08^b^
	TR10	66.15 ± 0.06^d^	33.85 ± 0.03^a^	434.90 ± 1.57^a^	478 ± 1.61^b^	1.26 ± 0.14^a^	0.22 ± 0.02^ab^
*p*-value		<0.0001	<0.0001	<0.0001	<0.0001	<0.2062	<0.0618
*F*-Value _3,11_		5477.5	3636.7	1551.2	1086.7	1.911	3.698
**J112**	Hmute	73.76 ± 0.06^b^	26.24 ± 0.03^c^	325.98 ± 1.76^c^	128 ± 1.09^c^	0.85 ± 0.04^a^	0.07 ± 0.02^a^
	Jorhat	71.30 ± 0.16^c^	28.70 ± 0.02^b^	225.49 ± 0.84^d^	311 ± 2.06^b^	0.67 ± 0.02^b^	0.05 ± 0.03^a^
	BC2-59	59.40 ± 0.02^d^	40.60 ± 0.13^a^	429.10 ± 0.64^b^	123 ± 2.12^c^	0.71 ± 0.02^b^	0.06 ± 0.02^a^
	TR10	84.17 ± 0.04^a^	15.83 ± 0.06^d^	476.57 ± 0.59^a^	325 ± 3.00^a^	0.86 ± 0.04^c^	0.07 ± 0.01^a^
*p-*value		<0.0001	<0.0001	<0.0001	<0.0001	<0.0054	<0.8910
*F*-Value _3,11_		13263	18982	10975	2618.4	9.358	0.2037


*The data are mean ± standard error (*n* = 3 observations). *p-*values were determined by one-way ANOVA with subsequent Tukey’s *post hoc* test. Values of treatment groups with different alphabetical letters show statistically significant difference at *p* < 0.05, and values with similar letters show no significance at *p* > 0.05. The *F*-value is the ratio of the mean regression sum of squares divided by the mean error sum of squares (variance within samples/variance between samples).*

## Discussion

*Bombyx mori* is a monophagous insect that feeds exclusively on mulberry leaf for its growth as well as accumulation of energy during non-feeding stages (larval, molting, spinning, pupal, and adult). The larvae need food that not only meets its nutritional requirements but must also be capable of being assimilated and converted into energy required for growth and development. The present work was performed to study the comparative analysis of different mulberry plant varieties to evaluate differences in their biochemical nutritive values and their physiological effect on different strains of *B. mori*. It is one of the most promising income sources and will involve less investment through better utilization of the resources with the knowledge of the plant nutrient profile and ecological suitability of the silkworm strains.

In the present study, analysis of biochemical composition of four different varieties of mulberry leaves showed that the levels of protein, carbohydrate, and reducing sugar were greater in the Hmute mulberry plant variety. The TR10 and Jorhat mulberry varieties showed significant amounts of calcium, potassium, magnesium and phosphorus, and in turn also performed well in terms of insect fibroin content, filament length and percentage of shell. Shinde et al. (2014) stated that silk productivity and quality are based on mulberry plant variety, its nutrients and climatic conditions. Quality of feed plays a remarkable role and is an important parameter used for evaluation for choosing suitable varieties for silkworm rearing as the nutrient quality of food plants, which affects its conversion into insect biomass, and this in turn affects the economic traits of cocoons (Das et al., 2001; Kumar and Vadamalai, 2010).

Nutritional efficiency study of all the bivoltine silkworm strains used in our study revealed variation in their nutritional requirements when reared with different host plants. Significant differences in growth rate (GR) were observed in most of the strains reared on four different host plants. High GR was observed in insects fed with Hmute, Jorhat, and TR10 mulberry plant variety leaves. CR was significantly higher in TR10 and Jorhat mulberry plant varieties for all the silkworm strains tested. Approximate digestibility (AD) is the indicator showing the amount of food consumed by the larva versus retention of food in the midgut, reflecting the effectiveness of digestive enzymes and ingestion of nutrients from the food. High AD means nutrients in the diet are ingested appropriately and converted into energy. Based on the value of the AD, it was observed that the TR10 mulberry plant variety is the best plant for digestibility in all the *Bombyx* strains tested. Silkworm nutrition is an important component in silk production, and this constituent determines the silk quality and silk trade all over the world. Low-quality mulberry plant feed significantly influences the performance of the life cycle process (growth of larva, developmental period, fecundity, silk production, and quality of silk) in terms of quality of leaf (Ramesha et al., 2010) and metabolic conversion of food into silk substance (Parra and Kogan, 1981; Paul et al., 1992; Levesque et al., 2002).

Significant improvement in larval growth characters were observed in larvae reared on high amino acid-containing mulberry plants (Radjabi, 2010). In the present study, similar results were obtained from our experiment – Hmute and TR10 mulberry plant variety-reared insects showed high growth rate due to the presence of high amino acid contents. There was a strong positive correlation between leaf moisture content and approximate digestibility (Radha, 2013), and in our study as well, most of the strains performed best on its preferred host plant TR10 mulberry plant variety. High food index values were observed in the chosen *B. mori* strains because of the high nutrient quality present in the TR10 mulberry plant variety. This observation corroborates the findings of Applebaum (1985) and Slansky and Scriber (1985), establishing the influence of insect digestion on the nutritional composition of a host. Rahmathulla et al. (2005) emphasized the association between mulberry varieties and food indices, especially ECD and ECI with insect biomass.

Assal et al. (1994) studied the nutritional behavior of *B. mori* with Fenoxycarb and reported the importance of food indices in terms of silk production. Kumara and Roy (2011) used food indices as a vital tool to identify profitable silkworm breeds. In our study, *B. mori* reared on TR10 and Jorhat mulberry plant varieties had higher mineral contents and consequently, consumption indices as well as approximate digestibility were found to be higher when compared to other food sources. Larvae fed with diet containing more minerals divert minimum energy for maintenance, by which the larvae can channel maximum energy for silk production. Yamamoto and Fujimaki (1982) and Sabhat et al. (2011) reported the alterations in food indices among the different breeds and same breed of *B. mori* when fed on the leaves of different nutritional quality.

Improvement in the leaf silk conversion ability of a given mulberry genotype or silkworm race will add to its economics (Trivedi and Nair, 1998). A large amount of data are available on the evaluation of mulberry varieties against silkworm rearing and economic parameters (Iwanari and Ohno, 1969; Thangamani and Vivekanandan, 1984; Das and Vijayaraghavan, 1990; Gangwar, 2011). In our study, significant differences were observed in larval parameters and commercial cocoon characters for all the silkworm strain reared with different mulberry varieties.

According to Fonseca et al. (1990) and Seidavi (2011), rearing success of silkworm races varied significantly even under the same conditions, with some of them being better performers, whereas some races showed poor performance. The present study also confirms the same where different host plants were evaluated for different silkworm strains. Gangawar (2010) found that among eight mulberry varieties screened for nutritional suitability, silkworm larvae fed on BR2 variety leaves showed increased larval weight and better economic traits in comparison to other varieties. In the present study, silk filament length of cocoons recovered from silkworms reared on different mulberry varieties falls within the range between 600 and 1500 m (FAO, 1999), and cocoons recovered from silkworms reared on Jorhat mulberry variety leaves produced the longest filament length. Cocoon and shell weights are the important parameters evaluated for productivity, and shell weight percentage is the amount of raw silk that is reeled from the cocoons and varies according to age and strain of silkworm (Gaviria et al., 2006).

Feeding with high content of potassium, magnesium and calcium in mulberry leaves significantly increased the shell percentage (Mane et al., 1998), which has been found in the larvae reared on TR10 and Jorhat plant varieties, which may in turn be due to the high amount of amino acids, potassium, magnesium, calcium present in the leaves (Verma and Atwal, 1963). Rearing performance of all the silkworm strains used in our study proved to be better when fed with Jorhat mulberry variety leaves followed by TR10. Among the different silkworm strains used, the CSR4 × CSR2 strain fed with the Jorhat mulberry variety exhibited the best performance. The FC2 × FC1 strain fed with the Jorhat mulberry variety proved promising. Besides the Jorhat mulberry variety, the FC1 × FC2 strain fed with the TR10 mulberry plant variety also showed good performance. These plant varieties supported superior growth and development of silkworm larvae, which is reflected in quality cocoon characteristics. From the present results, *B. mori* strains CSR4 × CSR2, FC1 × FC2, and FC2 × FC1 fed with Jorhat and TR10 mulberry plant varieties turn out to be superior among other *B. mori* strains as well as host plants used in this experiment.

Different strains of *B. mori* showed variations in fibroin percentage, hemolymph protein, silk gland protein, amylase and protease for different mulberry plant varieties. The conversion of host plant nutrients into silk protein mainly takes place during the larval stages, and protein metabolism is a major biochemical process that helps in characterizing different stages of development (Chen and Pan, 1996). Nutrition is linked to the physiology of digestion, and the ability of silkworms to secrete digestive enzymes is influenced by the nutrient profile of the diet (Kellner et al., 1887). The differences in enzyme activity, in our study, indicated that some silkworm strains fed with different host plants are more efficient in biomass deconstruction, while others are not. Activity of the enzyme amylase was high in those larvae reared with the Jorhat mulberry variety and the TR10 mulberry variety, which might be due to the sufficient amount of raw substrate resulting from high food intake. The proteolytic enzyme, proteases, are found in abundance in the late larval stages of silkworms, which aids the digestion of leaf fibrous proteins found in their coarse leaf diet (Ito, 1978). The proteolytic activity of the gut in relation to protein diet has been studied in many insects (Hamano and Mukaiyama, 1970). In the present study, protease activity was high in larvae fed with the Jorhat mulberry plant variety, and it is presumed that the diet may facilitate the enzyme to act on their substrate.

Hemolymph serves as a reservoir for nutrients and metabolites during metamorphosis. The cellular structures of the silk gland also differentiate and repair themselves for synthesis of silk proteins by utilizing the free amino acids present in the hemolymph (Mathur et al., 1989; Mahmoud et al., 2013). The silk is secreted by the silk glands, which are a reservoir for two silk proteins, fibroin and sericin (Zhang et al., 2006). In the present study, the silk gland contained more protein content when compared to hemolymph in the majority of silkworm strains used. *B. mori* produces twin threads of silk fibroin coated by a protective layer of sericin, and the silk protein is an essential constituent of cocoon filament (Komatsu, 1975). In our study, we have observed that fibroin percentage is significantly higher for most of the *B. mori* strains fed with the TR10 and Jorhat mulberry plant varieties, which may be due to the presence of high amino acid and carbohydrate levels. Additionally, hemolymph protein was significantly higher for TR10 mulberry plant variety-reared larva, followed by Jorhat mulberry plant variety-reared larva, which significantly increases the fibroin percentage in silk gland as the amino acids resulting from digestion are transported directly to the silk gland via hemolymph (Bricteux et al., 1965). Approximate digestibility was found high for the TR10 mulberry plant variety- followed by Jorhat mulberry plant variety-reared larva, maybe due to the presence of high amylase in the larva reared on TR10 and the Jorhat plant variety.

Silkworm requires specific nutrient components such as essential sugars, amino acids, proteins, and vitamins for its optimal growth and development (Sengupta et al., 1972; Khedr et al., 2013). Poor nutrition diets will directly affect the primary biochemical and physiological metabolism in insects, and in turn alter the detoxification system leading to increased susceptibility to diseases (Lindroth et al., 1991).

Sericulture industry can develop the rural economy of any state as it is a part of the tradition and culture of the local populace and hence is an eco-friendly production process with skilled households. It is one of the most promising income resources without spending much for its cultivation and better utilization with the knowledge of the plant nutrient sources and ecological suitability. The optimal levels of macronutrients in the host plants are sufficient for silkworm larval growth, whereas high levels of micronutrients are very much essential for better silk quality. The findings of the present study can form a platform for further research on silkworm physiology, especially under the hilly and high altitude agro-climatic conditions. From the results, we have observed that *B. mori* strains CSR4 × CSR2, FC1 × FC2, and FC2 × FC1 and mulberry varieties Jorhat and TR10 performed better in silkworm rearing tests under hilly tropical agro-climatic conditions. Such mulberry varieties and silkworm strains can be recommended for more field trials by farmers and can be used for sustainable growth and development of the sericulture industry.

## Author Contributions

LR, GG, BC, and NSK participated in the research design. LR and SG conducted the experiments. LR, SG, BC, TB, and NSK performed the data analysis. LR, SG, GG, TB, and NSK wrote or contributed to the writing of the manuscript. SS involved in major revision of the manuscript in both data analysis and rewriting the manuscript. All authors read and approved the final manuscript.

## Conflict of Interest Statement

The authors declare that the research was conducted in the absence of any commercial or financial relationships that could be construed as a potential conflict of interest.
