# Supplementary material for: Influence of Micronutrients on the Food Consumption Rate and Silk Production of Bombyx mori (Lepidoptera: Bombycidae) Reared on Mulberry Plants Grown in a Mountainous Agro-Ecological Condition
Source: Front Physiol. 2019 Jul 9;10:878. doi: 10.3389/fphys.2019.00878 (PMC6634293; doi:10.3389/fphys.2019.00878)
Supplement: Supplementary file 1 [file Data_Sheet_1.docx]

**WORKFLOW**

**Plantation of mulberry**

**Mass culture of Silkworm strains**

**Larval Food utilization indices**

**Silk worm larva performance on different diets**

**Biochemical estimation of different strains of *B. mori* reared on different mulberry varieties**

**Statistical Analysis for host plant preference and economic parameters for finding the best suited host plants**

**Supplementary Table 1: Mulberry host plant varieties used in the study**

| **MULBERRY VARIETIES (recommended for hilly regions)** |
| --- |
| **Local (H1) -** Locally available mulberry leaves  **Jorhat (H2)** - Origin not known, maintained at CSR & TI, CSB, Jorhat  **BC2-59 (H3)-** Developed at CSR & TI, CSB, Berhampore, by back crossing of hybrid of   Matigare Local x Kosen (Japanese variety) with Kosen twice.  **TR10 (H4)-** Triploid selection of Berhampore S-1 variety developed at CSR & TI,   Berhampore. |
|  |
|  |
|  |
|  |
|  |
|  |


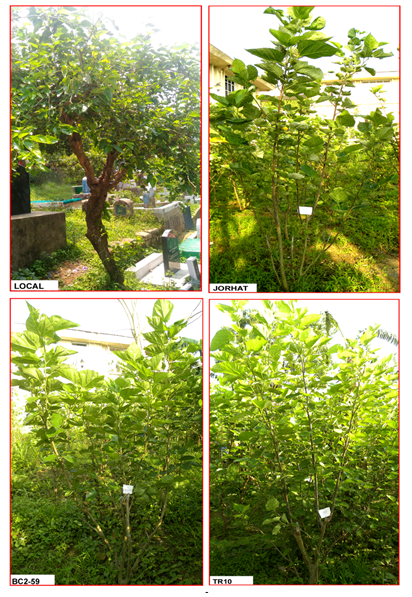


**Supplementary Figure 1: Silkworm strains (recommended for hilly regions) used in the study**


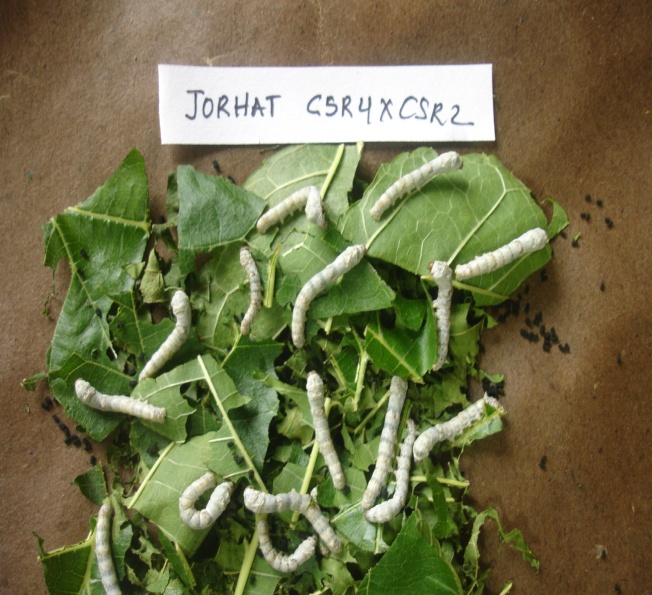

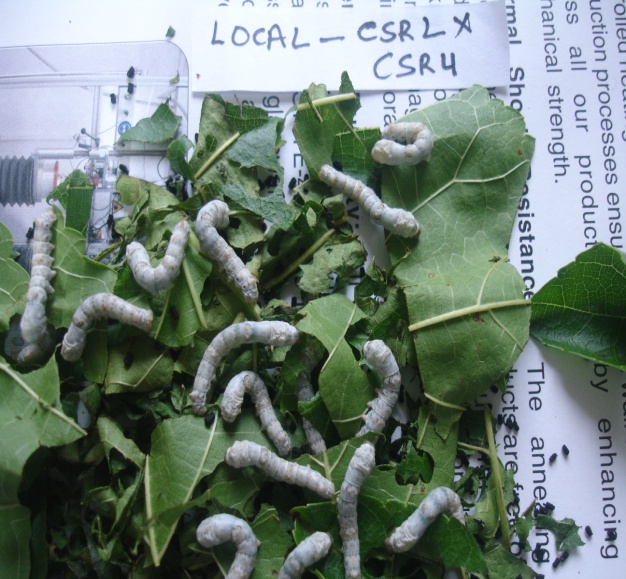


CSR4 X CSR2 CSR2 X CSR4


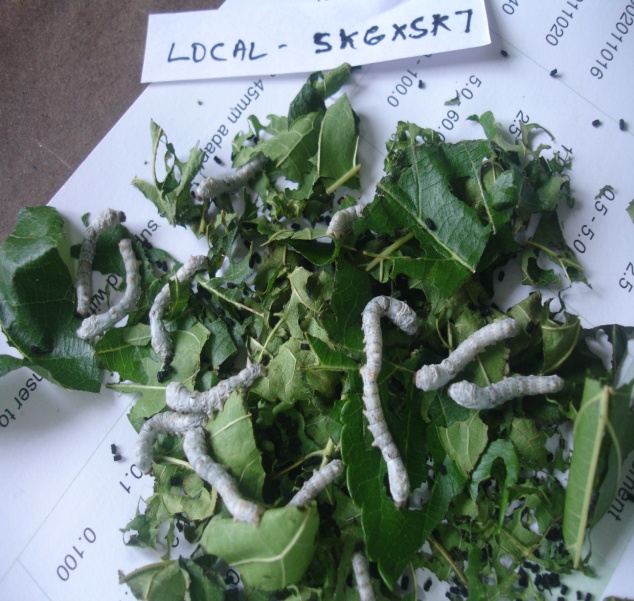

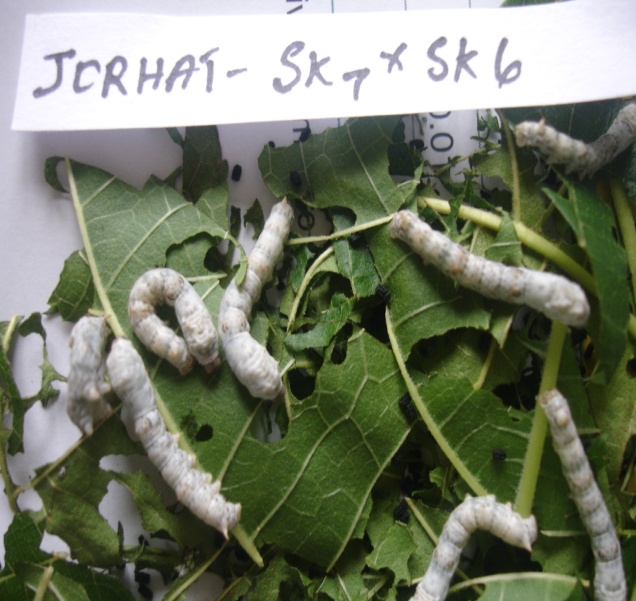


SK6 X SK7 SK7 X SK6


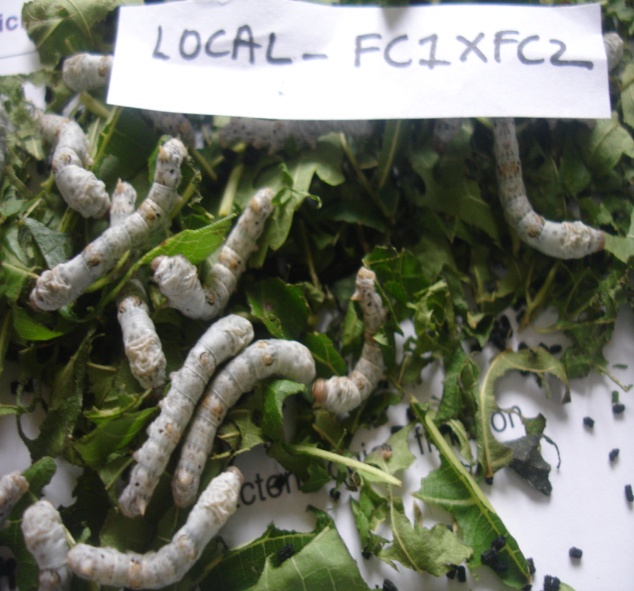

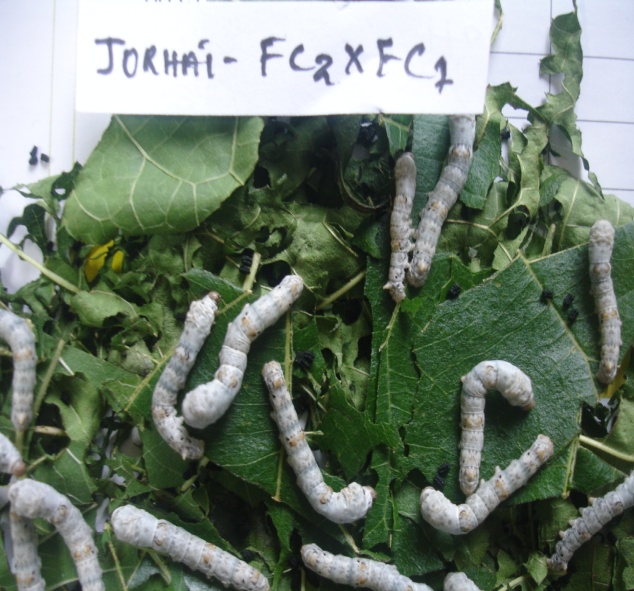


FC1 X FC2 FC2 X FC1


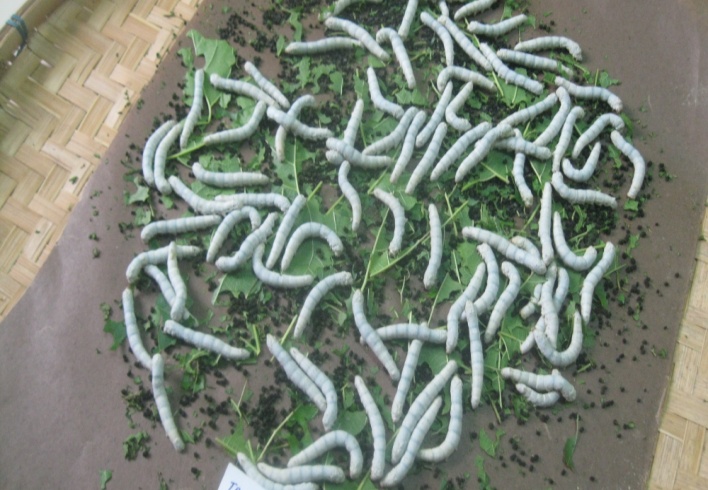


J112
